# Beneficial effects of berberine in the treatment of diabetes and its complications

**DOI:** 10.3389/fphar.2025.1701513

**Published:** 2026-01-29

**Authors:** Shanyao Liu, Jie Shen, Fanghui Xu, Lu Niu, Fengchan Wang, Guojing Zhao

**Affiliations:** 1 Qingdao Traditional Chinese Medicine Hospital (Qingdao Hiser Hospital), Qingdao, China; 2 School of Pharmacy, Qingdao University Medical College, Qingdao, China

**Keywords:** berberine, diabetes mellitus, herbal medicine, pharmacological activities, structural modification

## Abstract

Coptis chinensis (Huanglian), a key component in numerous classical Chinese herbal formulas, is traditionally applied for treating metabolic diseases based on its activity including clear heat, dry dampness, purge fire, and detoxify. Berberine (BBR), one key active component from Coptis chinensis, was contained in numerous classical Chinese herbal formulas for improving insulin resistance and regulating blood glucose levels, making them applicable for diabetes mellitus (DM) treatment. Clinical trials confirm that BBR monotherapy reduces glycated hemoglobin (HbA1c) by 1.5% in T2DM patients comparable to metformin. This review aims to explore its applications and current research progress in DM therapy. This article systematically reviews the modern separation, extraction, and purification techniques for BBR, its molecular pharmacological mechanisms, and advances in novel delivery technologies for diabetes treatment. This review synthesizes evidence that BBR exerts its anti-diabetic effects through multi-tiered mechanisms converging on the amelioration of insulin resistance and systemic inflammation. The findings provide a theoretical foundation for optimizing BBR’s clinical application and promote the transformation of traditional Chinese medicine from empirical usage to a scientific and standardized therapeutic paradigm. By integrating BBR’s multi-target pharmacology with cutting-edge delivery technologies, this review provides a transformative perspective, positioning BBR not merely as a natural product but as a scaffold for the rational development of next-generation, multi-target diabetes therapeutics.

## 1 Introduction

Diabetes mellitus (DM) is characterised by chronic hyperglycaemia. Currently, approximately 350 million people worldwide are affected by diabetes, with about 5% having Type 1 diabetes (T1DM), and 95% of those diagnosed with Type 2 diabetes (T2DM). The former is an autoimmune disease, marked by specific destruction of pancreatic β cells responsible for insulin secretion by T lymphocytes, leading to an absolute loss of insulin production (American Diabetes Association Professional Practice Committee, 2024). The characteristics of T2DM are insulin resistance (where the body cells are insensitive to insulin) and the associated relative insufficiency of insulin secretion (Yang et al., 2023).

According to the World Health Organization, approximately 70% of the global population relies on medicinal plants for disease treatment, with over 1,200 plant species evaluated for their potential in diabetes management (Abid et al., 2025). In classic anti-diabetic formulas such as Gegen Qinlian Decoction, Coptis chinensis (Huanglian) serves as a “minister herb,” with its key active compound berberine (BBR) showing remarkable efficacy (Zhang et al., 2025). Berberine can be used to treat various diseases such as diabetes, non-alcoholic fatty liver disease, cancer and cardiovascular diseases (CVD) (Asghari et al., 2025). Recent systems pharmacology studies reveal that BBR alone modulates multiple diabetes-related targets. These include the brain-gut axis, gut microbiota-short-chain fatty acids (SCFAs), farnesoid X receptor (FXR), and peroxisome proliferator-activated receptor alpha (PPARα), improving insulin resistance (IR), lowering blood glucose, regulating lipid metabolism, and suppressing inflammation and oxidative stress, which establishes its therapeutic potential as an independent monomeric compound (Zhao et al., 2021a; Chen et al., 2023a; Utami et al., 2023).

Severe seminal reviews have comprehensively summarized the progress in the efficacy of berberine in the treatment of diabetes. While these prior reviews have been instrumental, they did not sufficiently discuss the signal pathway of berberine in the treatment of diabetes. This review systematically discusses BBR’s application in traditional formulas, its natural sources, extraction/isolation techniques, and therapeutic roles in diabetes and its complications. Additionally, it summarizes the advantages of BBR derivatives in diabetes treatment. The study aims to establish a theoretical foundation for further exploration of BBR’s pharmacological applications and structure-activity relationships, ultimately promoting BBR as a promising, safe, and effective therapeutic agent.

Although the above preclinical and clinical research results are encouraging, there are still several significant challenges and controversies in developing BBR into a standardized and highly effective anti-diabetic drug. The oral bioavailability of BBR is extremely low (Wang et al., 2017), and there is inconsistency among different clinical trials and animal studies. Moreover, there is still debate regarding the core target of BBR’s action, and its multi-target characteristic brings complexity to the mechanism elucidation while also offering therapeutic advantages. This review not only systematically summarizes the beneficial effects of BBR but also objectively examines these controversial results and existing challenges, and conducts in-depth discussions on novel strategies (such as structural modification and new drug delivery systems) aimed at overcoming these obstacles, with the aim of providing a more comprehensive and dialectical perspective for the in-depth research and clinical translation of BBR.

## 2 Literature search strategy

This narrative review aimed to provide a comprehensive overview of the current understanding of “Beneficial effects of Berberine in the treatment of diabetes and its complications”. To identify relevant literature, we conducted searches in the PubMed, Web of Science electronic databases and so on. The search strategy utilized key terms such as “Diabetes mellitus”, “Berberine”, and their related synonyms. The search was primarily focused on articles published between January 2020 and December 2025, with an emphasis on seminal works and high-impact reviews in the field. The inclusion of studies was based on their relevance to the core themes of this review, prioritizing original research and authoritative consensus statements. Given the narrative nature of this review, a formal systematic approach or adherence to PRISMA guidelines was not employed, allowing for a more flexible and interpretive synthesis of the vast and heterogeneous literature on this topic.

## 3 Application of BBR in antidiabetic therapy in Chinese herbal compound

**FIGURE 1:**
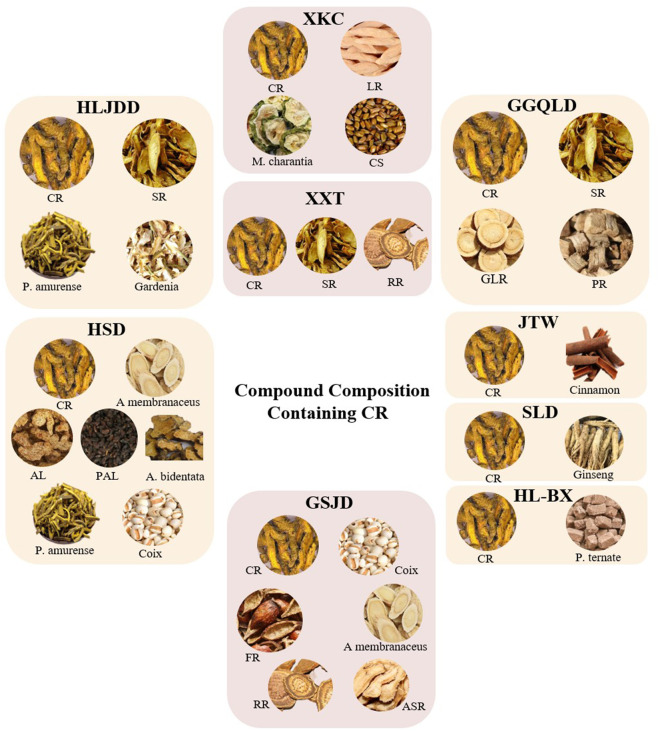
Traditional Chinese medicine formula containing BBR for diabetes. *Coptidis Rhizoma* (CR), *Scutellaria Radix* (SR), *Phellodendron amurense Rupr* (P. amurense), *Gardenia jasminoides J. Ellis* (Gardenia), *Liriopes Radix* (LR), *Momordica charantia* (M. charantia), *Cassiae Semen* (CS), *Pueraria Lobatae Radix* (PR), *Rhei radix et rhizome* (RR), *Glycyrrhizae Radix* (GLR), *Pinellia ternate* (P. ternate), *Coix lacryma-jobi* (Coix), *Rosa laevigata Michx* (FR), *Astragalus membranaceus* (A membranaceus), *Angelicae Sinensis Radix* (ASR), *Atractylodes lancea* (AL), *Plantago asiatica L* (PAL), *Achyranthes bidentata Blume* (A. bidentata).

Coptidis Rhizoma (Huanglian), a core component in traditional Chinese medicine (TCM) formulations for DM, exerts therapeutic effects by targeting the pathogenic “Yin deficiency with dryness-heat” syndrome (Xiaoke) in TCM theory. Its bitter-cold properties specifically clear stomach fire and alleviate cardiac irritabilit, addressing the hallmark symptoms of middle-Jiao heat excess, such as polyphagia, polydipsia, and restlessness. When combined with Yin-nourishing and Qi-tonifying herbs (e.g., Rehmanniae Radix and Ginseng Radix), this combinatorial strategy achieves simultaneous symptom relief and disease-modifying effects through synergistic “heat-clearing and Yin-preserving” mechanism, thereby delaying DM progression. Gegen Qinlian Decoction (GQD) alleviates hepatic endoplasmic reticulum stress-induced unfolded protein response and apoptosis, increases intracellular calcium ion levels, downregulates phosphorylated JNK (p-JNK), activates the IRS1/PI3K/Akt signaling pathway, improves insulin sensitivity, and regulates hepatic glycogen metabolism both *in vivo* and *in vitro* (Wang et al., 2020). Further analysis using UPLC identified four primary components in GQD: BBR, puerarin, baicalin, and liquiritin (Zhang et al., 2025). Tissue distribution and pharmacokinetic studies reveal that BBR predominantly accumulates in the colon with a mean retention time of 4.5–6.3 h, highlighting its therapeutic potential for diabetes-related intestinal disorders (Li et al., 2024a; Lu et al., 2022). Similarly, Shenlian (SL) decoction and Huangqi Simiao Decoction (HSD) exert hypoglycemic and intestinal protective effects by modulating the metabolism and diversity of gut microbiota (Sun et al., 2022). Additionally, Huanglian-Banxia (HL-BX) Decoction regulates brain-gut neurotransmitters via the MAPK signaling pathway, reducing food intake while accelerating gastric emptying and increasing body weight, making it a promising treatment for diabetic gastroparesis (Chen et al., 2024a). Furthermore, Huang-Lian-Jie-Du decoction (HLJDD) medicated serum significantly reduces IL-1β secretion and NLRP3 inflammasome activity through Atg7-mediated autophagy, demonstrating protective effects on the BV2 microglial cell line (Tian et al., 2024). Modern studies have revealed that BBR, the primary active component in Coptis chinensis, and its mechanism of regulating glucose and lipid metabolism are just scientific confirmation of the theory of “clearing heat to restore qi transformation” in TCM. Traditional Chinese medicine formula containing BBR for diabetes is shown in Figure 1.

However, berberine undergoes extensive metabolism after oral administration and its plasma concentration is extremely low (Wang et al., 2017). This is because BBR, after absorption from the gastrointestinal tract, is widely distributed in various organs, but its concentration in the blood is low. After oral administration of BBR(200 mg/kg) in rats, studies indicates that BBR was quickly distributed in the liver, kidneys, muscle, lungs, brain, heart, pancreas and fat in a descending order of its amount. And BBR’s level in most of studied tissues was higher (or much higher) than that in plasma 4 h after administration (Tan et al., 2013). The BBR that is absorbed into the body can be converted into a variety of metabolites, and most of the BBR and its metabolites remain in gastrointestinal tract and are eventually excreted from the body with feces. In the form of BBR and major metabolites, 22.83% of administered dose was recovered from bile, urine, and feces. A large number of BBR was found in feces with a recovered rate of 22.74% after dosing in 48 h (Ma et al., 2013).

## 4 Source, extraction and isolation of BBR

**FIGURE 2:**
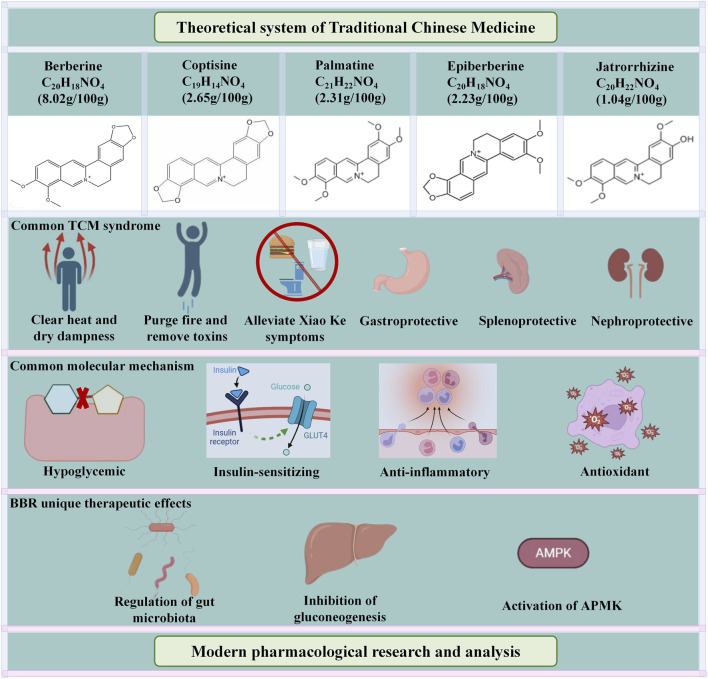
Main components of *Rhizoma coptidis* against DM.

Rhizoma coptidis, herbaceous plant from the Ranunculaceae family, is first recorded in the “Shen Nong Ben Cao Jing” (Panigrahi and Mohanty, 2023). It is commonly used medicinally for its rhizome, which is extremely bitter in taste and has a cold nature (Wang et al., 2019a). It possesses a wide range of pharmacological activities, including anti-cancer, anti-inflammatory, antiviral, antioxidant, anti-hepatic steatosis, anti-diabetes, and anti-arrhythmia (Xie et al., 2022). Notably, the anti-diabetic effect of Rhizoma coptidis was first documented by Tao Hongjing in 1,500 years ago (Feng et al., 2019). At present, there are more than 100 chemical constituents isolated from Rhizoma coptidis, including alkaloids, lignans, flavonoids, and acidic components. Alkaloids are the primary pharmacodynamic components, with BBR and coptisine mainly contributing to the hypoglycemic effects, while palmatine, jatrorrhizine, and epiberberine play distinct synergistic roles (Li et al., 2024b) (Figure 2). In addition, BBR is also the most abundant (5%–8%) and representative component in Rhizoma coptidis (McCubrey et al., 2017). It is worth noting that the content of alkaloids in Rhizoma coptidis collected in different seasons did not change much, but the content of alkaloids in different parts of Rhizoma coptidis varied greatly, with higher alkaloid levels found closer to the main root of Rhizoma coptidis. Moreover, the content of alkaloids remains almost unchanged after washing in fresh products, whereas it is greatly reduced in dried products after washing (Pang et al., 2014). In Table 1, we have summarized the traditional Chinese medicine compound formulas, including their main components, mechanisms of action and therapeutic effects.

**TABLE 1:** Summary table of traditional Chinese medicine compound formulas, their main components, mechanisms of action and therapeutic effects.

Formula name (Chinese Pinyin)	Key active components	Primary proposed mechanisms	Major outcomes/effects
Gegen Qinlian Decoction (GQD)	Berberine, Puerarin, Baicalin, Liquiritin	• Reduces hepatic ER stress and apoptosis • Activates IRS1/PI3K/Akt signaling pathway • Modulates hepatic glycogen metabolism • Inhibits hepatic ferroptosis by targeting Nrf2	Improves insulin sensitivity and reduces blood glucose
Huanglian Banxia Decoction (HL-BX)	Berberine	Modulates brain-gut neurotransmitters via the MAPK signaling pathway	Reduces food intake, accelerates gastric emptying; potential treatment for diabetic gastroparesis
Huang-Lian-Jie-Du decoction (HLJDD)	Berberine	Inhibits NLRP3 inflammasome activity and reduces IL-1β secretion via Atg7-mediated autophagy	Exhibits hypoglycemic and intestinal protective effects
Shenlian Decoction (SL)	Berberine	Modulates the metabolism and diversity of gut microbiota	Exhibits hypoglycemic and intestinal protective effects
Processed Coptis with Evodia	Berberine (content varies with processing method)	Information missing (Processing alters herbal properties according to TCM theory)	Alters BBR content and intestinal absorption; used for different syndromes (e.g., liver fire, stomach fire) based on processing
Huang-Gui Solid Dispersion (HGSD)	Berberine, Sodium Decanoate (absorption enhancer)	• Inhibits the iPLA2/p38 MAPK pathway to reduce β-cell apoptosis • Reduces GR/PI3K association to improve insulin resistance	5-fold increase in BBR bioavailability; significantly improves glucose and lipid metabolism in diabetic rats
Poly-Herbal Extract (PHE)	Berberine (6.442%)	Increases fecal short-chain fatty acids (SCFAs) and beneficial gut flora	Lowers fasting blood glucose (FBG)

### 4.1 Commonly used extraction methods for BBR

There are numerous methods for extraction of BBR, but extraction rates of different methods are quite different. More importantly, there are no uniform criteria to calculate extraction yields, which hinders comparability. It is recommended that prior to extraction, the content of BBR in Rhizoma coptidis should be determined according to pharmacopoeia standards, and then the extraction rate can be calculated as (the mass of extracted BBR/the mass of BBR contained in Rhizoma coptidis × 100%). The commonly used extraction methods and parameter settings for BBR are summarized in Table 2.

**TABLE 2:** Extraction methods and condition parameters of BBR.

Extraction method	Condition Settings	Yield	Advantage	Limitations	References
Water extraction					
​	Used 12 volumes of aqueous solution and reflux extraction for 1 h	4.61%	Stable and feasible Environmentally friendly	Low extraction yield	McCubrey et al. (2017)
Acid hydrolysis					
​	Soaked Rhizoma coptidis in 0.4% dilute sulfuric acid 1 day in advance and extracted them with 16 volumes of dilute sulfuric acid	4.61%	​	​	Huang JiaWei and Sheng ZhenHua (2011), Guoping and Song (2009)
	Used 12 times the solvent content and 5 mL·min^-1^ percolation rate	11.61%	Easy operation Mature process High extraction yield	Environmental pollution Corrosion equipment	Wang et al. (2010a)
	Cold soaking with 0.2% sulfuric acid for 72 h followed by salting out with 15% NaCl	—	​	​	Wang et al. (2010b)
	Used 0.2% H_2_SO_4_ solid-liquid ratio was 1 ∶ 35 and Tween 80 solid-liquid ratio was 2 ∶ 3	10.10%	​	​	Zhifang and He (2009)
Alcohol extraction					
​	Refluxing extraction twice at 85 °C for 2 h with 8 volumes of 50% ethanol solution	7.70%	Stable process Simple equipment High extraction yield	Long extraction time Large amount of extraction solvent used	Yan and Tianjiao (2008)
	Refluxing extraction twice at 62 °C for 1 h with 11 volumes of 53% ethanol solution	8.00%			Wu et al. (2007)
	Extracted with 13 volumes of 50% ethanol solution extracted three times for 1 h each time	6.10%			Gao et al. (2012)
	Extracted with 9 volumes of 60% ethanol by reflux for 3 times for 1 h each time	—			Xin et al. (2013)
Desorption internal boiling					
​	Dissolved with ethanol, and then a certain amount of boiling hot water was added as the extractant	10.12%	Fast extraction speed High active ingredients Less ethanol consumption	​	Yuan et al. (2011)
Ultrasonic					
​	Sonication with 80% ethanol solution at 50 °C for 30 min	8.38%	Short extraction time Wide range of application	​	Zhongxing et al. (2014)
Microwave					
​	Heating the ratio of material to liquid at 1: 30 for 10 min under microwave oven high fire	9.25%	Mild and efficient	​	Huang and Zhou (2006)
	Extracted with 25 volumes of 50% ethanol solution by microwave reflux twice for 10 min at 80 °C	8.50%	High purity Safety and environmental protection	​	Lin et al. (2013)
Enzyme extraction					
​	Reacting with 30 mg/g cellulase at pH 4.0 and temperature 40 °C for 90 min	0.75%	Mild and efficient High extraction yield	​	Binliang and Zhou (2006)
Liquid membrane					
​	Set the pH of mother liquor to 10, the concentration of hydrochloric acid in the membrane to 0.3 mol/L, the ratio of oil to 5, and the ratio of milk to water to 1:4	2.50%	Facilitates isolation and enrichment	​	Guoping and Song (2009)

#### 4.1.1 Water extraction method

BBR is a quaternary ammonium base and is slightly soluble in water Huang JiaWei and Sheng ZhenHua (2011) used 12 volumes of aqueous solution and refluxed extraction for 1 h. The final extraction yield was 4.61%. Extraction of BBR with water is stable and feasible, and dodn’t pollute the environment. However, the extraction rate is low, which is not conducive to full use of medicinal materials. Experiments also indicated that prior addition of lime milk could dissolve BBR in water in its free state, which was then converted into hydrochloride precipitate, thereby increasing the extraction efficiency (Guoping and Song, 2009).

#### 4.1.2 Acid hydrolysis method

Acid hydrolysis method takes advantage of the high solubility of BBR sulfate in water and the near-insolubility of its hydrochloride salt to achieve separation and extraction, making it one of the most commonly used methods in industry. Wang et al. soaked the *Rhizoma coptidis* granules in 0.4% dilute sulfuric acid solution 1 day in advance and extracted with 16 times the volume of dilute sulfuric acid, achieving a final extraction rate of 4.61%. This method is simple to operate, uses readily available materials, and has a mature process, laying the foundation for the industrial production of BBR (Wang et al., 2010a; Wang et al., 2010b). Cen et al. demonstrated through single-factor and orthogonal experiments that when the *R. coptidis* granules were coarsely ground, the solvent volume was 12 times, and the permeation rate was 5 mL·min^-1^, the extraction rate reached 11.61%. The method is simple to operate and has a high extraction rate, which avoids the heat loss of BBR hydrochloride, but it is time-consuming (Zhifang and He, 2009). Guo et al. showed that BBR obtained by cold soaking with 0.2% sulfuric acid for 72 h followed by salting out with 15% NaCl had the best extraction yield. Xu et al.’s results showed the highest extraction rates using 0.2% H2SO4 (solid-liquid ratio of 1∶35) and Tween 80 (solid-liquid ratio of 2∶3) (Yan and Tianjiao, 2008). Because the addition of Tween increases the extraction rate by 33.9%, this has great guiding significance for industrial production. However, acid hydrolysis method is easy to cause environmental pollution and equipment corrosion. Therefore, it is often combined with other methods in practical applications.

#### 4.1.3 Alcohol extraction method

The results showed that 7.7% BBR extraction rate could be obtained by refluxing extraction twice with 8 volumes of 50% ethanol solution (Wu et al., 2007). The method is simple and high extraction rate, which is suitable for industrial production. It had also been shown that 8.0% BBR extraction yield could be obtained by refluxing extraction twice with 11 volumes of 53% ethanol solution. In addition to removing heavy metal cadmium, this method improved the quality of medicinal materials and increased market competitiveness (Gao et al., 2012). Liu et al. showed that 6.1% BBR extraction yield could be obtained with 13 volumes of 50% ethanol solution extracted three times for 1 h each time (Xin et al., 2013). Xu et al. showed that when the pulverization degree of medicinal materials was 10 mesh, they were extracted with 9 volumes of 60% ethanol by reflux for 3 times for 1 h each time (Yuan et al., 2011). This method saves energy consumption and resources, had low requirements for production equipment, and is suitable for the extraction of single herbs and prescription preparations of Rhizoma coptidis.

#### 4.1.4 Desorption internal boiling method

On the basis of alcohol extraction, the powder of *R. coptidis* was dissolved with ethanol, and then a certain amount of boiling hot water was added as the extractant, so as to accelerate the extraction rate (Zhongxing et al., 2014). Compared with the traditional alcohol extraction method, the time is shortened by 10 times and the extraction rate is obviously increased.

#### 4.1.5 Ultrasonic method

In recent years, ultrasonic technology has shown great advantages in extraction of natural products. It uses ultrasonic radiation to destroy medicinal material cells and promote the dissolution of cell contents, so as to achieve the goal of rapid extraction of natural products at low temperature. Huang and Zhou (2006) showed that 8.38% BBR extraction rate could be obtained by sonication with 80% ethanol solution for 30 min, which was 42% higher than traditional ethanol extraction method. The method only use ethanol as extraction solvent and the extraction time was short, it’s convenient for wide application in industrial production.

#### 4.1.6 Microwave method

Microwave is a new extraction method developed in recent years. Xu et al. showed that heating for 10 min under microwave oven high fire (ratio of material to liquid 1:30) increased the extraction rate of BBR by 42.2%. Lin et al. refluxed twice in a microwave with 25 volumes of 50% ethanol solution, the extraction yield and purity of BBR were 8.5% and 73.2%, respectively (Lin et al., 2013). A simple and efficient extraction and purification process by microwave method is established, which laid a foundation for further industrial production. In addition, microwave pretreatment combined with Soxhlet process is often used, which can maintain the higher concentration difference of BBR components inside and outside the matrix material during the extraction process, thereby improving the extraction efficiency.

#### 4.1.7 Soxhlet extraction method

The Soxhlet extraction (SE) technique applied for the extraction of berberine from B. lycium, which employs the principle of solvent reflux and siphoning to continuously extract the solid pure solvent, conserving solvent and achieving high extraction efficiency. Rapid determination and novel optimisation method for berberine extraction has been developed by Soxhlet extraction utilising central composite design-response surface methodology (CCD-RSM). And the highest yield of 13.39% was obtained under the conditions of extraction time of 7.28 h, ethanol concentration of 52.21%, and solvent-to-sample ratio of 21.78 mL/g. This leads to higher extraction efficiency in berberine yield.

#### 4.1.8 Other extraction methods

**FIGURE 3:**
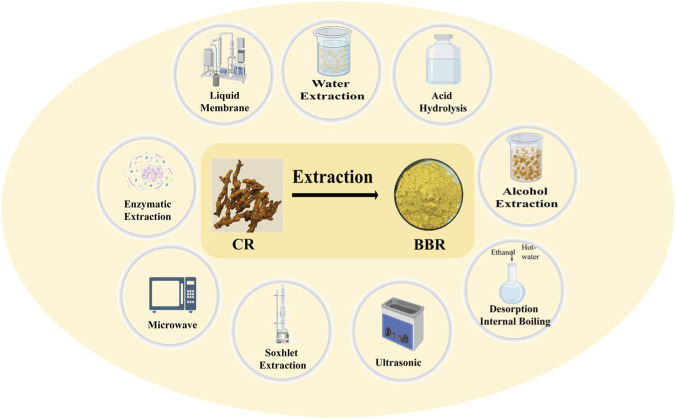
Common BBR extraction methods.

Because plant cell walls are mainly composed of cellulose, cellulases can catalyze the cleavage of cellulose β-D-glucosidic bonds and destroy plant cell walls, promoting the leaching of plant active ingredients. The results showed that 0.752% BBR extraction rate could be obtained by reacting with 30 mg/g cellulase for 90 min, which was 49% higher than that by traditional ethanol extraction (Binliang and Zhou, 2006). Xi et al. used different components of *R. coptidis* to set the pH of mother liquor to 10, the concentration of hydrochloric acid in the membrane to 0.3 mol/L, the ratio of oil to 5 and the ratio of milk to water to 1:4 to obtain a good separation and enrichment of BBR (Guoping and Song, 2009). Therefore, we summarize the extraction and processing methods of BBR (Figure 3; Table 2).

### 4.2 Processing methods for BBR

#### 4.2.1 Effect of different processing methods on BBR content

*Rhizoma coptidis*, known for its cold and bitter properties. The typical processing methods include treatments with ginger juice, vinegar, and pig bile (Wang et al., 2024). After processing with ginger juice, the BBR content increases significantly (Yuan et al., 2019), roasting with ginger also enhances BBR levels (Wang et al., 2018). In addition, the source of ginger has little effect on the content and quality of BBR, but the heating mode may be the key factor. When processed with pig bile, *R. coptidis* promotes the dissolution of BBR, enhancing its cooling effect. Yan et al. processed *R. coptidis* with ginger, evodia rutaecarpa, rice wine, vinegar, and bile, the contents of BBR were higher after processing (Yang et al., 2014). On the contrary, some studies had shown that the content of BBR in *R. coptidis* was significantly reduced after processing with evodia rutaecarpa juice. More importantly, there are significant differences in BBR content in *R. coptidis* prepared from different doses of evodia rutaecarpa, with 7.5 mL evodia rutaecarpa juice having the highest BBR content (Yang et al., 2014). Therefore, the dosage of evodia rutaecarpa should be determined according to the processing requirements to achieve the optimal results.

#### 4.2.2 Effect of different processing methods on BBR function

In the text “Ben Jing Feng Yuan,” it is stated for treating heart fire, use it raw. For addressing the deficiency fire of the liver, fry it with vinegar. For the stagnation of liver fire with qi stagnation, fry it with the juice of evodia rutaecarpa. It can be seen that the processing of Rhizoma coptidis is closely related to its efficacy. Wang et al. studied the effects of different processing methods on BBR’s content and intestinal absorption. The content of BBR gradually decreased according to the order of vinegar Rhizoma coptidis, ginger Rhizoma coptidis, evodia rutaecarpa Rhizoma coptidis, raw Rhizoma coptidis, wine Rhizoma coptidis, fried Rhizoma coptidis. However, the order of intestinal absorption from strong to weak was wine Rhizoma coptidis, vinegar Rhizoma coptidis, evodia rutaecarpa Rhizoma coptidis, ginger Rhizoma coptidis, raw Rhizoma coptidis, salt Rhizoma coptidis, and fried Rhizoma coptidis (Wang et al., 2011). In addition, it had also been shown that BBR content gradually decreased according to the order of ginger Rhizoma coptidis, wine Rhizoma coptidis, evodia rutaecarpa Rhizoma coptidis and raw Rhizoma coptidis, and the antibacterial effect was enhanced after processing (wine Rhizoma coptidis > ginger Rhizoma coptidis > Evodia rutaecarpa Rhizoma coptidis > raw Rhizoma coptidis) (Chengyan et al., 2022).

In addition, four drying methods (direct sunlight, covering with thin paper under sunlight, stir-frying with slight fire, oven baking) had also been studied to compare the effects on BBR content. Covering with thin paper is a traditional method to dry Rhizoma coptidis decoction pieces. The relationship between the content of BBR and different drying methods is shown as follows: covering drying with thin paper under sunlight (5.78%) > oven drying (5.73%) > stir-frying under slight fire (5.60%) > direct drying under sunlight (5.50%) (Liu et al., 2025). In practice, the operation of tissue paper covering require a lot of manpower and material resources. Although the content of BBR in oven drying is lower than that in tissue paper covering drying, the difference between them is small. It is important to ensure that the appearance and color remain unchanged while greatly shortening the drying time. It is worth noting that with the increase of temperature (>170 °C), BBR would be converted into berberrubine, thus reducing the content. This is due to the destruction of certain structures in BBR due to excessive temperatures, which generate new chemical constituents (Zhong et al., 2023). In summary, different processing and drying methods have a certain degree of impact on the content, absorption and function of BBR, which provides a scientific basis for clinical application and lays a solid foundation for further research.

### 4.3 Critical evaluation of BBR extraction and separation technology and prospects of green technology

Each of the above extraction methods has its own characteristics, but their drawbacks in terms of solvent consumption, environmental impact, and long-term operating costs prompted us to search for more sustainable and cost-effective alternatives. Although Water extraction method, Acid hydrolysis method and Alcohol extraction method are all suitable for industrial production (Huang and Zhenhua, 2011; Zhifang and He, 2009; Gao et al., 2012), however, it also involves the problems of low extraction rate, easy corrosion of equipment, safety and environmental protection. Heavy use of volatile organic solvents (e.g., petroleum ether, chloroform, benzene, etc.) may cause air pollution and health risks to workers. The large amount of acidic or organic waste liquid in the production process and the high cost of subsequent treatment will also increase the burden on the environment. Prolonged extraction can also cause corrosion of the equipment (Huang and Huang, 2025).

However, for the emerging green extraction technology, Ultrasonic method uses ultrasonic cavitation effect to break plant cells and improve mass transfer efficiency. The equipment is simple and the energy consumption is low (Huang and Zhou, 2006). Microwave method has industrial microwave extraction equipment, the technology is relatively mature, easy to integrate with the existing production line (Lin et al., 2013). It is one of the most promising green technologies for large-scale application. The enzymatic extraction method has a mild effect, but the enzymatic cost is high, the reaction time is long, and the process conditions (pH, temperature) are strictly controlled, so the cost-effectiveness in large-scale production needs to be improved (Binliang and Zhou, 2006).

The extraction technology of berberine is developing towards greener and more efficient. Future research should focus on optimizing the process parameters of these green technologies, developing economically feasible solvent recycling systems, reducing waste generation as well as environmental hazards during extraction, and conducting a comprehensive life cycle assessment to drive the true realization of green manufacturing of berberine.

## 5 Beneficial effects of BBR in the treatment of diabetes

**FIGURE 4:**
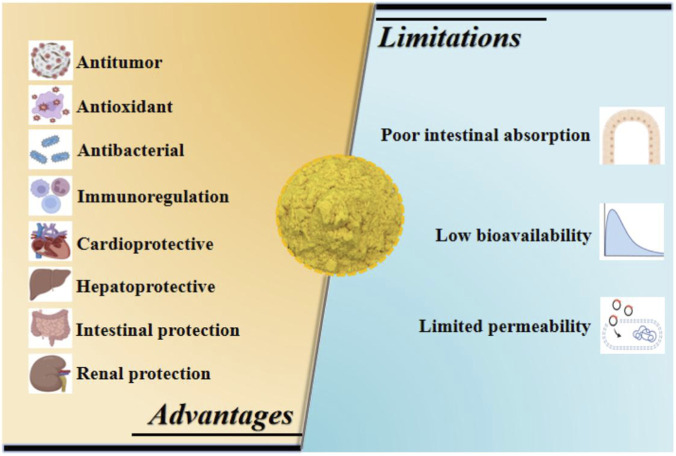
Common pharmacological advantages and limitations of BBR application.

Figure 4 illustrates the common pharmacological effects and limitations of the BBR application.

### 5.1 BBR enhances insulin secretion

Within the pancreas, there exist several types of endocrine cells: α, β, δ and pancreatic polypeptide cells. Among these, β cells are responsible for the secretion of insulin, which is crucial for maintaining glucose homeostasis (Hou et al., 2020). Sirtuin 1 (SIRT1), a member of the sirtuin family of enzymes, can modulate various physiological activities of pancreatic β cells. Lv et al. found elevated expression levels of islet miR-204 in a diabetic model, accompanied by decreased SIRT1 protein levels. BBR treatment could reduce the levels of miR-204 and increase the expression of SIRT1. TargetScan predicted a high degree of complementary binding between miR-204 and SIRT1 (Lv et al., 2021). Glucagon-like peptide-1 (GLP-1), released by intestinal L cells in a glucose-dependent manner, is an insulinotropic hormone that plays a significant role in regulating glucose metabolism (Tadaki et al., 2019). It has been reported that BBR exerted its anti-diabetic effects by alleviating oxidative stress and mitochondrial dysfunction as well as promoting the secretion of GLP-1 (Yang et al., 2024a). Study used berberrubine (500 mg/kg body weight) or palmatine (500 mg/kg body weight) or the equivalent amount of solvent via gastric gavage to mouse 1 h prior to glucose load. Then OGTT was performed and about 50 μL of blood was taken from the angular vein at the first four time points (0, 15, 30, and 60 min) for the determination of GLP-1 and insulin levels. They facilitated GLP-1 secretion and enhanced glucose tolerance in mice *in vivo*. Furthermore, hepatocyte nuclear factor 4α (HNF4α) is another nuclear transcription factor that plays a very important role in the pathogenesis and progression of DM (Firdous et al., 2022). Studies have suggested that BBR has the potential to become an insulin secretagogue, which may be attributed to the upregulation of HNF4α expression (Zhao et al., 2021b).

Ferroptosis is a non-apoptotic form of cell death that is involved in the pathogenesis of T1DM. BBR can stimulate the expression of glutathione peroxidase 4 (GPX4), decrease the levels of Fe2+ and reactive oxygen species (ROS), thereby inhibiting ferroptosis in pancreatic β cells (Dang et al., 2023). Additionally, BBR increases oxygen consumption and thermogenesis to combat the decline in systemic metabolism, reduces fat content in the offspring of mice with gestational diabetes, and reverses hyperinsulinemia (Cole et al., 2021a). Single-walled carbon nanotubes (SWCNT) activated oxidative stress pathways in pancreatic islets. Excessive oxidative stress decreases insulin secretion and accelerate the progression of diabetes (Panigrahi and Mohanty, 2023; Caturano et al., 2023; Lee and Lee, 2022) Pretreatment with BBR and its nanoparticles was able to reduce SWCNT-induced increases in ROS levels while enhancing insulin secretion (Golfakhrabadi et al., 2023). Recent studies had shown that BBR directly targets TP53-induced glycolysis and apoptosis regulator (TIGAR) protein to attenuate the conversion of fructose-2,6-diphosphate to fructose-6-phosphate and exerted a strong hypoglycemic effect (Qi et al., 2025).

### 5.2 BBR ameliorates IR

IR is a physiological condition where cells fail to respond to insulin despite insulin levels in the blood remain high level, which is a characteristic feature of T2DM (Kong et al., 2024). Clinical studies have shown that daily oral BBR can significantly improve blood glucose and IR in diabetic patients, and no serious adverse effects as well as nephrotoxicity or hepatotoxicity have been detected. HIMABERB® 500 mg was given three times daily to the treatment group, which dosing regimen is typical dosing of 0.5–1.5 g/day in trials treating diabetes mellitus (Panigrahi and Mohanty, 2023). Several studies have demonstrated that BBR can ameliorate IR through various mechanisms.

#### 5.2.1 Protein kinase C-insulin receptor (PKC-InsR) signaling pathway

Overproduction of glucocorticoids (GC) induces obesity and IR by enhancing glucocorticoid receptor (GR) activation, which plays a critical role in the progression of T2DM (Xu et al., 2020; Lu et al., 2020). The combination of BBR and Huang-Gui solid dispersion (HGSD), a preparation of sodium carbonate, could enhance the bioavailability of BBR, improve IR by reducing the association between GR/GRα and PI3K, and restore blood glucose and GC levels in skeletal muscle (Meng et al., 2020). Therefore, PKC-InsR signaling pathway is considered to be a critical pathway for improving IR.

#### 5.2.2 Retinol binding protein 4-glucose transporter 4 (RBP4-GLUT4) system

RBP4 is a protein synthesized in liver and adipose tissue, and its expression level is closely associated with T2DM. Studies involving the specific deletion of GLUT4 in adipose tissue of mice have shown a strong correlation between RBP4, obesity, and T2DM (Askari et al., 2023). Furthermore, the levels of RBP4 in serum are inversely correlated with GLUT expression in adipose tissue, and elevated RBP4 levels impair insulin signaling in muscle and increased hepatic glucose output (Gasmi et al., 2024). These results suggested that RBP4 may serve as an effective biomarker to predict IR and T2DM, and reducing serum RBP4 levels may be an effective strategy to prevent and treat T2DM. In addition, BBR also ameliorated glucosamine hydrochloride (Glcn) -induced IR and increased GLUT2 expression in a PPARγ/FGF21-dependent manner which reduced T2DM induced hepatic lipid accumulation and pancreatic injury (Chen et al., 2023a).

#### 5.2.3 AMP-activated protein kinase (AMPK) signaling pathway

AMP-activated protein kinase (AMPK) is a serine/threonine protein kinase that plays a crucial role in regulating cellular metabolism and energy homeostasis, and is considered a potential therapeutic target for the treatment of DM (Entezari et al., 2022; Park et al., 2023; Steinberg and Hardie, 2023). In IR models, it has been demonstrated that BBR could significantly improve insulin sensitivity by activating the AMPK signaling pathway (Utami et al., 2023; Dong et al., 2021). Oxyberberine (OBB), an important metabolite of BBR, specifically binds hemoglobin, upregulates HO-1 expression in diabetic rats, and improves IR by activating the phosphoinositide 3-kinase/protein kinase B (PI3K/Akt) and AMP-activated protein kinase B (AMPK) pathways, which lays the foundation for the translational application of BBR (Dou et al., 2022). Superoxide dismutase (SOD) is an antioxidant enzyme that maintains the redox balance within living organisms. During the onset of DM, the function of SOD is impaired (Demir et al., 2021). After treatment with BBR, the upregulation of SOD expression plays a crucial role in antioxidant activity (Yuan et al., 2023).

#### 5.2.4 Hypoxia-inducible factors (HIF) -2α signaling pathway

HIFs participate in the physiological activities of various cells under both hypoxic and normoxic conditions. Increasing evidence suggested that HIF plays a critical role in regulating IR, obesity, T2DM, and nonalcoholic fatty liver disease (NAFLD) (Catrina and Zheng, 2021). In T2DM, IR and abnormal lipid metabolism exacerbate hepatic hypoxia. In turn, the increase in ceramide levels under hypoxic conditions further accelerates the progression of IR. BBR aims to reduced IR by downregulating the expression of HIF-2α target genes, inhibiting the PP2A-AKT-GSK3β(23) (He et al., 2022a).

*In vitro*, primary hepatocytes pretreated with TNF-α were utilized to assess the effect of BBR on hepatic insulin sensitivity. The results indicated that BBR could attenuate ERK1/2-induced phosphorylation of insulin receptor substrate (IRS)-1 serine residues, thereby enhancing IRS-1 tyrosine phosphorylation and Akt activation (Shakeri et al., 2024). Molecular docking revealed that BBR could effectively bind to MEK1/2. Additionally, due to the structural similarity between MEKK1 and MEK1/2, MEKK1 was also considered a target of BBR. These results offer new theoretical grounds for the application of BBR in the treatment and prevention of T2DM (Li et al., 2022a).

#### 5.2.5 Silent information regulator 1 (SIRT1)/OpticAtrophyType1 (Opa1) signaling pathway

In a palmitic acid (PA) induced hepatocyte IR cell model, the deficiency of Opa1 often leaded to an imbalance in mitochondrial fusion/fission, impairing the insulin signaling pathway. After treatment with BBR, the expression of Opa1 was increased, which improved mitochondrial function (Guo et al., 2023). Similarly, in diabetic animal models, BBR treatment could also enhance the SIRT1/Opa1 signaling pathway, mitigating hepatic IR (Xu et al., 2022).

#### 5.2.6 Peroxisome proliferator activated receptor (PPAR) signaling pathway

BBR ameliorates IR in HepG2 cells by modulating PPAR signaling pathway in KEGG enrichment analysis and free fatty acid-induced insulin resistant HepG2 cell model (IR-HepG2) (Chen et al., 2024a). It has also been shown that BBR is able to upregulate ADPN, IRS2, PI3Kp85, p-Akt (Ser473)/Akt, p-mTOR (Ser2448)/mTOR, PPARα and CPT1α levels and downregulate p-GSK3β (Ser9)/GSK3β, ChREBP, SREBP-1C, ACC1 and FASN levels, and it is speculated that BBR regulates BMAL1-centered clock metabolic network and improves IR in HepG2 cells (Ahmad et al., 2025).

### 5.3 BBR ameliorates DM by modulating disturbances in glucose and lipid metabolism

Metabolic disorders (e.g., dyslipidemia) are closely related to DM, and studies have shown that BBR has a significant regulatory effect on metabolic disorders, including affecting the function of pancreatic β-cells, regulating the levels of cholesterol and triglycerides in the blood, and promoting fecal lipid excretion (Nazari et al., 2024; Panigrahi and Mohanty, 2023; Zhu et al., 2019; Yang et al., 2019). Aldose reductase is an enzyme essential for the reduction of glucose to sorbitol, and the application of aldose reductase inhibitors can alleviate various symptoms of DM (Bi et al., 2022; Gopal et al., 2023). Glycogen phosphorylase (GP), the rate-limiting enzyme in glycogen degradation, is overexpressed in DM mice. BBR treatment is able to reduce the level of GP in the liver, while reducing its affinity for glycogen, slowing glycogen degradation, and improving glucose homeostasis (Liu et al., 2020). At the same time, BBR also enhances hepatic glycogen synthesis and improves hepatic insulin sensitivity by increasing SIRT1 expression (Sui et al., 2021) as well as accelerating intracellular cAMP degradation (Zhong et al., 2020). The serine/threonine kinase (Akt) is involved in the regulation of downstream factors of glycogen synthesis and is a key mediator of glucose and lipid metabolism (Li et al., 2022b; Tian et al., 2023; Chen et al., 2022). Research indicates that a significant part of BBR’s hypoglycemic effect is due to the restoration of Akt activity (Elkomy et al., 2022). BBR is able to enhance the concentrations of NO and cGMP and activate the NO/cGMP/PKG signaling pathway to inhibit gluconeogenesis in hepatocytes by activating the AKT1/MAPK axis (Li et al., 2023a). In addition, BBR can activate the LKB1-AMPK-TORC2 pathway, reducing gluconeogenesis in skeletal muscle and adipose tissue (Zhang et al., 2020; Jiang et al., 2015).

The anti-inflammatory pathway mediated by acetylcholine is crucial in the treatment of diabetes. Acetylcholinesterase (AChE) is an enzyme of acetylcholine, and its overexpression can accelerate the progression of diabetes. Following BBR treatment, it inhibits acetylcholinesterase activity and decreases the expression of pro-inflammatory cytokines (IL-1β and TNF-α), thereby improving IR and glucose metabolism (Cao et al., 2022). He et al. used the hydrogen bonding interaction between pioglitazone and BBR to significantly ameliorate IR and glucose/lipid metabolism in diabetic mice (Qian et al., 2022). Studies have also suggested that BBR’s regulatory effect on lipid metabolism may be related to the expression of carnitine palmitoyltransferase 1a (Piao et al., 2017; Jia et al., 2024). BBR improves mitochondrial swelling in the liver and intestine of mice, thereby inhibiting lipid metabolism and alleviating obesity and fatty liver. In the study on diabetes rats, the best dose of BBR to improve lipid metabolism is 156 mg/kg per day (Yu et al., 2021).

### 5.4 BBR relieves DM through anti-inflammatory mechanisms

#### 5.4.1 BBR modulates gut microbiota as an anti-inflammatory mechanism

Studies have demonstrated that changes in gut microbiota can trigger a series of inflammatory responses, while IR and T2DM are closely related to persistent inflammatory states (Cheng et al., 2022). BBR (6.442%), as the main component of natural multi-herb Chinese herbal formula (PHE), could increase the content of fecal short-chain fatty acids (SCFA) and intestinal flora and decrease the level of FBG (Singh et al., 2024). Moreover, modulation of gut microbiota by BBR contributed to alleviate the inflammatory state of DM. This is because the gut microbiota convertes BBR into absorbable dihydroBBR (dhBBR) and increases the intestinal absorption rate (He et al., 2022b). BBR alone or in combination with stachyose improves glucose metabolism, increases gut microbiota richness, and regulates fecal metabolomics in diabetic rats (Zhao et al., 2021a; Lyu et al., 2022). Although BBR increases the abundance and diversity of gut microbiota, transplantation of fecal microbiota from BBR-treated mice into normal mice does not alter the metabolism of recipient mice. This is due to that BBR relieves obesity mainly by inhibiting mitochondrial complex I in the gut and liver of mice, and this process is not affected by gut microbiota. In addition, in terms of gene expression, BBR combined with miR-10a-5p treatment is also able to inhibit the inflammatory response and alleviate systemic glucose tolerance (Li et al., 2021). In the treatment of BBR combined with probiotics, it is able to synergistically reduced postprandial lipids (PL) and achieved better lipid control in T2DM (Zhang et al., 2020; Li et al., 2022c). In addition, BBR also increases GLP-2 secretion, downregulated inflammatory factors (TLR-4, NF-kB, and TNF-a), and restores intestinal barrier function (Wang et al., 2021a). However, some gastrointestinal side effects have also been observed during BBR treatment. Subsequent genomic and metabolomic studies have shown that this may be associated with inhibition of deoxycholic acid (DCA) biotransformation by Ruminococcus bromidus (Shu et al., 2021).

Treatment with BBR has increased the microbial community dominated by Bacteroidetes and non-pathogenic Clostridia, establishing unique gut microbiota profile and bile acid (BA) characteristics. Correlation analysis indicates that changes in BA were closely associated with improvements in markers associated with T2DM. Li et al. showed that BBR upregulated TGR5 expression and glucagon-like peptide secretion in colonic tissue of diabetic mice and improved gut microbiota and energy metabolism (Li et al., 2020). In addition, it can also regulate the expression of genes related to energy metabolism in gut microbes, and BBR changes the level of tryptophan metabolites, increases the species diversity and uniformity of gut microbes reduces the level of aromatic amino acids, and increases the content of probiotics after being metabolized by oxidation, demethylation, and hydrogenation processes *in vivo* (Chen et al., 2023b). Excess carbohydrates are not conducive to fish growth. Similarly, it has also been shown that the addition of BBR to a high-carbohydrate diet can change the proportion of tilapia gut microbiota to stimulate the synthesis of bile acids, promote glycolysis, inhibit gluconeogenesis, and achieve the purpose of maintaining blood glucose stability, which will facilitate the research and development of high-carbohydrate diets in aquaculture (Liu et al., 2024). These findings suggestes that the anti-diabetic activity of BBR is at least in part achieved by modulating the structure of the gut microbiota and the composition of BAs, making the regulation of gut microbiota a promising target for the management of diabetes.

#### 5.4.2 BBR modulates inflammatory response by MAPK signaling pathway

The MAPK signaling pathway, composed of p38 MAPK, c-Jun N-terminal kinase (JNK), and extracellular signal-regulated kinase (ERK), is involved in cellular proliferation, differentiation, and migration (Li et al., 2022d). p38 MAPK is closely associated with inflammation, glucose uptake and apoptosis. BBR, acting as an inhibitor of p38 MAPK, can reduce the phosphorylation of related inflammatory factors (Li et al., 2022d). Helper T cells (Th) 1 and Th17 are inflammatory T cell types that play a significant role in the pathogenesis of T1DM. BBR can also treat T1DM by activating ERK1/2 to inhibit the differentiation of Th17 cells and blocking the activation of p38 MAPK and JNK to inhibit the differentiation of Th1 cells (Cui et al., 2009).

#### 5.4.3 BBR modulates inflammatory response by NF-κB signaling pathway

T2DM is a condition characterized by low-grade inflammation. The NF-κB signaling plays a pivotal role in inflammatory and immune responses, and IκB kinase β (IKKβ) is a key regulator of NF-κB activation (Huang et al., 2023). BBR suppressed the inflammatory response and decreases the expression of intercellular adhesion molecule-1, transforming growth factor-β1 and fibronectin through IKKβ or NF-κB dependent mechanisms (Dang et al., 2023). It is accompanied by a decrease in cytokine content in metabolic cells, immune cells, as well as pancreatic β-cells, such as tumor necrosis factor-α, IL-6, IL-1β, monocyte chemoattractant protein-1, and inducible nitric oxide synthase (Lan et al., 2022). In a gestational diabetes (GDM) rat model, BBR decreases nuclear translocation of IKKβ and NF-κB p65 in rat liver tissue, as well as phosphorylation levels of JNK, IRS-1, and AKT in liver tissue, decreasing glycogen synthesis capacity (Elkomy et al., 2022). Research also indicates that combined application of BBR and genistein could effectively lower the fasting blood glucose levels in fasting rats through targeting antihyperglycemic and NF-κB regulatory pathways, and alleviate excessive inflammation (Alkholifi et al., 2023).

### 5.5 BBR relieves DM by exerting antioxidant activity

Oxidative stress reflects an imbalance between the production and elimination of reactive oxygen species (ROS). Excessive ROS can lead to the damage and apoptosis of pancreatic β cells, thereby reducing insulin secretion. Studies have shown that BBR could inhibit oxidative stress and ameliorate kidney damage (Zhao et al., 2023), pancreatic dysfunction (Cole et al., 2021a), and cognitive impairment (Bertoncello et al., 2024). It also regulates mitochondrial energy metabolism under hyperglycemic conditions by activating the C/EBPβ/Gas5/miR-18a-5p and C-PGC-1α signaling pathways, inhibiting the production of ROS and apoptosis (Xu et al., 2021a).

BBR attenuates nicotinamide adenine dinucleotide phosphate (NADPH) oxidase. NADPH oxidase is a major source of ROS, which activation is closely associated with the development of DM, obesity and atherosclerosis. Therefore, NADPH oxidase is considered a potential target for the treatment of diabetes (Moon, 2023). BBR inhibits the overexpression of NADPH oxidase and reduces ROS production in macrophages and endothelial cells under inflammatory conditions (Paul et al., 2019).

And it can be clearly seen from Section 5.2.3 that BBR mitigates oxidative stress by regulating the AMPK signaling pathway.

### 5.6 Other mechanisms of BBR against diabetes

**FIGURE 5:**
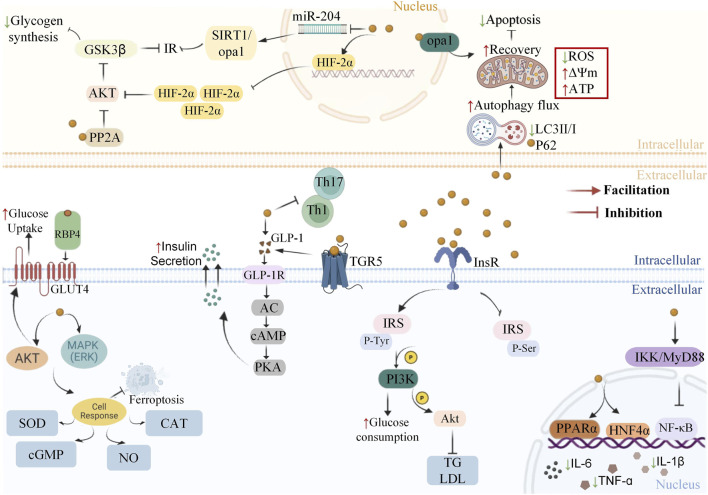
Potential pathways through which BBR exerts protective effects in diabetes treatment.

Some studies suggest that BBR may function as a glucokinase activator or insulin sensitizer to stimulate the release of insuline. It is also able to significantly reduce the activities of sucrase and α-glucosidase in the small intestine (Chen et al., 2023a). Excessive binding of leukotriene B4 (LTB4) to its receptor BLT1 induce chronic inflammation and exacerbate IR. BBR, on the other hand, targetes BLT1 and regulates the LTB4-BLT1 axis to alleviate IR and inflammation (Gong et al., 2021). Figure 5 summarizes potential pathways which BBR exerts protective effects in diabetes treatment.

## 6 Beneficial effects of BBR in the treatment of diabetic complications

DM could lead to a variety of complications such as encephalopathy, hypertension, neuropathy, retinopathy, nephropathy, as well as memory impairment (Deng et al., 2023). BBR treatment was able to improve a variety of impaired mechanisms, but the details of this need to be further explored.

### 6.1 BBR alleviates diabetic bone disease

Diabetic osteopathy is considered a unique disease that primarily affects the feet. A prominent feature of bone resorption is the “curling” of patients’ toes, which can cause significant discomfort and disability. *In vitro* model of diabetic osteopathy, BBR has been shown to alleviate the inhibitory effects of high glucose on bone formation by upregulating the IRS-1 signaling pathway mediated by ROS (Shao et al., 2021). However, some research indicates that although BBR has certain effects on the microstructure and bone formation markers of the tibial diaphysis in diabetic rats, it did not improve bone mineralization and biomechanical properties (Londzin et al., 2022). Based on the multi-target therapeutic effects of BBR, it alleviates diabetic tendinopathy by activating autophagy in tendon cells, reducing tendon cell damage and inflammatory responses (Zhu et al., 2022). There is evidence that BBR improves lipid and glucose metabolism through the Smad pathway, reducing the expression of myostatin and increasing skeletal muscle mass (Liu et al., 2022). In studies of alveolar bone regeneration in diabetic patients, BBR restores autophagic flux, inhibits mitochondrial dysfunction, and promotes osteogenic differentiation, showing a good potential to promote alveolar bone remodeling in T2DM rats. The Ber@SF/PCL electrospinning nanofibrous membrane with a 2.5% loading concentration demonstrated the best bone regeneration effect (Ming et al., 2024). Moreover, BBR is also able to inhibit M1 polarization in the periodontitis microenvironment, regulate the balance between M1/M2, and alleviate alveolar bone loss in periodontitis models by inhibiting the NF-κB pathway (Xia et al., 2024). BBR have a certain effect on bone quality, but appropriate repair strategies should be developed according to the purpose of treatment when applied.

### 6.2 BBR alleviates diabetic retinopathy

Diabetic retinopathy is the primary cause of DM onset and the foremost reason for newly acquired blindness. Thus, finding novel medications to treat diabetic retinopathy is of utmost importance. Insulin intervention can stimulate the activity of HIF-1α and VEGF in retinal endothelial cells.

Diabetic retinopathy (DR) is one of the major complications of DM, thus attracting increasing interest in novel therapeutic agents for its treatment. BBR inhibits the activation of retinal endothelial cells in a time- and dose-dependent manner through the Akt/mTOR/HIF-1α/VEGF pathway, thereby improving diabetic retinopathy (Wang et al., 2021b). Additionally, by regulating glucose and lipid metabolism and suppressing the HIF-1α/VEGF/NF-κB pathway, it reduces the accumulation of retinal glycogen and levels of inflammatory factors, protecting the retina from damage induced by high glucose levels (Yin et al., 2021). The regulation of inflammatory factors in the retina by BBR, on the one hand, is due to the fact that BBR directly inhibits the expression of the transcription factor RORγt and promotes the expression of the transcription factor Foxp3 in T cells, resulting in a downregulation of the Th17/Treg ratio. On the other hand, BBR also inhibits TNF-α, IL-1β, and IL-6 secretion by dendritic cells (Tang et al., 2024). Through differential protein analysis, Gene Ontology (GO) enrichment and Kyoto Encyclopedia of Genes and Genomes (KEGG) pathways, proteomics with four-dimensional independent data collection (4D-DIA), and molecular docking experiments, it is shown that the therapeutic effects of BBR on diabetic retinopathy involved rRNA processing, ribosome generation, and phospholipid binding. It is also able to exert protection against retinal pigment epithelial cells (RPEs) by decreasing carbonic anhydrase 1 (CA1) expression (Na et al., 2023). Notably, BBR may be a more beneficial agent compared to fenofibrate and rosiglitazone (Utami et al., 2023). It has fewer adverse reactions compared to these synthetic drugs. Retinal advanced glycation end products (AGEs) formation and activation of AGEs-related signaling pathways also contribute to the development of retinopathy. A low dose of BBR inhibits the occurrence of diabetic retinopathy through the inhibition of AGE/RAGE signaling in the retina. A high dose of BBR directly reduces blood glucose levels, suppresses subsequent AGE formation, and improves overall diabetic symptoms (Wang et al., 2021c). Furthermore, BBR treatment reduces the activity of aldose reductase and exerts a positive therapeutic effect on lens lesions in diabetic rats (Zych et al., 2020). It is also able to improve the survival rate of retinal ganglion cells and improve visual function through the GABAAR/PKC-α pathway (Fang et al., 2022). Therefore, BBR represents a highly promising treatment strategy for diabetic retinopathy. By directly intervening in the specific pathways of retinal lesions and improving systemic metabolic disorders, it provides a solid scientific basis for the development of new therapies for this complication.

### 6.3 BBR alleviates diabetic neuropathy

Approximately 60% of DM patients will experience neuropathic pain. Moreover, multiple acute attacks of neuropathy are associated with inflammatory levels (Baum et al., 2021). BBR is widely used to treat DM-induced neuropathy and enhances cognitive function due to its significant anti-inflammatory effects. In DM patients, fluctuations in blood glucose levels lead to lipid metabolism disorders, which further increase molecular levels associated with oxidative stress, such as malondialdehyde (MDA), lipid peroxides, and reduced antioxidant molecules. BBR treatmented reversed these adverse effects and showed significant neuroprotective and antioxidant potential (Adefegha et al., 2021; Wu et al., 2024). It also effectively downregulated the abnormal phosphorylation of Aβ and tau protein and reduced apoptosis of hippocampal neurons (Zhang et al., 2021a). However, Zhang et al. showed that low-dose BBR only altered metabolic abnormalities in diabetic mice and did not showed significant neuroprotection (Zhang et al., 2021b). Higher doses of BBR may be required to alleviate diabetes-related cognitive impairment (Xu et al., 2021b).

In a model of cognitive decline associated with diabetes, BBR treatment decreases levels of inflammatory factors such as IL-6, iNOS, and TNF-α, increases acetylcholine levels and permeability of the blood-brain barrier, thereby improving cognitive performance in mice (Gupta et al., 2025). BBR activates the Keap1/Nrf2/ARE pathway, upregulates the expression of antioxidant enzymes, and reduces cell damage, oxidative stress, and mitochondrial dysfunction in high glucose-injured neural strain cells PC12 (Yuan et al., 2023). In a rat model of diabetic depression-like behavior, the combination of BBR and ginsenoside (Rb1) improves glucose metabolism and IR, increases the expression of brain-derived neurotrophic factor protein, and relieves depression-like behavior in rats. Daily intragastric administration of BBR (150 mg/kg, combined with 20 mg/kg Ginsenoside Rb1) to rats for four consecutive weeks significantly upregulates BDNF protein expression in the hippocampus (Zhang et al., 2021c). In addition, BBR in combination with Rb1 or cinnamon improves glucose and lipid metabolism and IR, increases brain-derived neurotrophic factor protein expression, and alleviates depression-like behavior in animal models of diabetic depression-like behavior (Tang et al., 2024). Under electrical field stimulation, BBR promoted acetylcholine release by affecting calcium channels and also improved fundic nerve dysfunction to some extent (Hou et al., 2023). It could be seen that BBR alone or in combination has great clinical value in the treatment of diabetic neuropathy patients (Zhang et al., 2022a).

### 6.4 BBR alleviates diabetic kidney disease

Diabetic nephropathy (DN) is a unique complication that arises during the progression of Diabetes Mellitus (DM), affecting the glomerular region of the kidney. Globally, approximately 50% of end-stage renal diseases are attributed to DM (Xie et al., 2022). Urinary iron concentration, serum ferritin and hepcidin levels were increased and total antioxidant capacity was decreased in DN rats, and BBR may reverse these adverse effects by improving iron overload and oxidative stress and also decrease the expression of renal fibrosis markers induced by DN (Sun et al., 2022). In addition, glomerular mesangial cells (GMCs) proliferate abnormally during DN disease progression, and BBR is able to inhibit the PI3K/Akt/AS160/GLUT1 signaling pathway and regulate high-glucose-induced cell cycle arrest in GMCs (Ni et al., 2022). In addition, protection of renal proximal tubular cells (NRK- 52E) may be associated with Sirt1-FoxO3a-Bnip3y-induced mitophagy (Saxena et al., 2024). Moreover, BBR regulates the metabolic shift from fatty acid oxidation to glycolysis, reducing lipid deposition in renal tubular epithelial cells and alleviating renal tubulointerstitial damage (Rong et al., 2021). It also suppress the NLRP3 inflammasome to inhibit the transformation of epithelial mesenchymal transition (EMT) and renal fibrosis induced by high glucose levels (Ma et al., 2022). It has also been shown that BBR is able to inhibit the expression of DNMT1 and DNMT2, as well as prevent methylation of the KLF4 promoter to upregulate KLF4 expression, reduce oxidative stress and expression of ferroptosis markers, rescue renal function in mice with diabetic nephropathy, and prevent renal fibrosis. Berberine protected the renal tissue structure in diabetic nephropathy mice in a dose-dependent manner. Intragastric administration of 200 mg/kg/day BBR to mice for eight consecutive weeks significantly improved renal function indicators and reduced renal fibrosis in a dose-dependent manner (Cai et al., 2024). Zhang et al. showed that both metformin and BBR alone or in combination improved IR and reduced progression of DN. Mechanistically, BBR primarily promoted the expression of Trib1, enhancing the renal protective effects of metformin, and ultimately inhibited the activation of fatty acid synthase and the NF-κB signaling pathway, while regulating lipid degradation and suppressing inflammatory responses, achieving better anti-DNF effects (Zhang et al., 2021d). *In vitro*, BBR activated autophagy in podocytes through the mTOR/P70S6K/4EBP1 signaling pathway, enhancing the expression of LC3II/LC3I and the number of autophagosomes, alleviating podocyte apoptosis, and exerting renal protection (Zhang et al., 2021d). Under high glucose conditions, lncRNA LOC102549726 was highly expressed in podocytes of DN rats. BBR inhibits migration and apoptosis of podocytes in DN by targeting the LOC102549726/EGF/FOXO1 axis (Wang et al., 2025). In conclusion, the existing evidence indicates that BBR effectively delays the progression of diabetic nephropathy by interfering with the core pathological processes such as oxidative stress, fibrosis, metabolic disorders, and abnormal cell proliferation and death. This constitutes the pharmacological basis for its renal protective effect.

### 6.5 BBR ameliorates diabetic cardiovascular disease

Diabetes is a major independent risk factor for the occurrence of atherosclerosis, characterized by hyperglycemia, hyperinsulinemia, and dyslipidemia (Tannu et al., 2024; Edgar et al., 2021). BBR demonstrates significant regulation of lipid and glucose metabolism, suggesting its potential to ameliorate atherosclerosis in diabetes. In both *in vitro* and *in vivo* experiments, BBR stimulates the expression of KLF16 and PPARα, ameliorates lipid and glucose metabolic disorders, and inhibits vascular inflammation (Man et al., 2022). BBR also significantly inhibits the production of C-reactive protein and inflammatory factors (IL-6, TNF-α), and increases adiponectin levels in rats. Moreover, after intervention, thoracic aorta contraction is reduced and relaxation response to SNP is enhanced in T2DM rats, suggesting that the protective effect of BBR on diabetic macrovascular complications is related to inhibition of inflammation and intervention of potassium channels. At the same time, BBR inhibits proliferation and migration of vascular smooth muscle cells and delays luminal narrowing. It also reduces the contraction of the thoracic aorta to phenylephrine and exert a protective effect against diabetic macrovascular complications (Wu et al., 2021). In the treatment of diabetic cardiomyopathy, BBR blocks inflammasome activation through the miR18a3p/Gsdmd pathway, attenuates pyroptosis, and improves biomarkers of cardiac function. Further studies show that BBR inhibition of pyroptosis is achieved by modulating the mTOR/mitochondrial reactive oxygen species (mtROS) axis (Zhong et al., 2024). After the combined intervention of BBR and fenugreek seeds in diabetic patients, fasting insulin and glycated hemoglobin levels were significantly reduced, enhancing the cardiac metabolic function in diabetic patients (Nematollahi et al., 2022; Cortez-Navarrete et al., 2023). In addition, BBR treatment increase the expression of enzymes involved in phospholipid and fatty acid uptake in the heart, which has a strong protective effect against cardiac dysfunction in gestational diabetic mice (Cole et al., 2021a; Cole et al., 2021b).

### 6.6 BBR ameliorates diabetic wound healing

Diabetic wound healing is hindered by infection, inflammation and oxidative stress, IL-17 signaling pathway plays an important role in the above links. The application of IL-17A inhibitors can accelerate wound healing. BBR also show significant IL-17 inhibition, enhancing the expression of vascular-related proteins (CD31, PDGF-BB, and ANG1), and accelerating diabetic wound healing with BBR (hfdSTZ + BBR, 0.038 mg/cm^2^) once a day (Zhang et al., 2022b). Hydrogel dressings prepared by mixing BBR and fungal polysaccharide with Carbomer in different ratios show excellent biocompatibility and significant antibacterial, anti-inflammatory and antioxidant effects in diabetic wound models. Moreover, BBR inhibit oxidative stress and apoptosis by activating TrxR1 and inhibiting its downstream JNK signaling pathway (Zhou et al., 2021). A hydrogel prepared from chitosan and BBR, with good stability and sustained release behavior, promoting wound healing by inducing angiogenesis and fibroblast proliferation (Panda et al., 2021). Hydrogel dressings prepared by mixing Bletilla striata polysaccharide (BSP)/silk cellulose (SF) and berberine (BER) also have good biocompatibility as well as antibacterial, anti-inflammatory and antioxidant properties, significantly accelerating the healing of diabetic wounds (Hu et al., 2023; Maity et al., 2022). In addition, loading BBR into other dressings did not affect the original physical properties of the dressings (Yin et al., 2022), but enhanced their antimicrobial properties and biological activity (Samadian et al., 2020). In order to further improve the bioavailability of BBR, the researchers encapsulated BBR in F127 micelles and designed an injectable ferrocene-cyclodextrin self-assembled oxidation-supramhydrogel drug delivery system to achieve high-quality diabetic wound healing (Liang et al., 2025).

### 6.7 BBR ameliorates diabetic liver damage

T2DM can also lead to liver cirrhosis and progress to non-alcoholic steatohepatitis (a form of fatty liver disease associated with IR). In NAFLD, aldo-keto reductase 1B10 (AKR1B10) is shown to be a target protein for BBR, which significantly improve hepatic steatosis and IR and decreased TG levels *in vivo* by targeting AKR1B10-mediated PPAR signaling pathway (Yang et al., 2024b). BBR has also been shown to improve lipid metabolism and reduce oxidative stress and hepatic steatosis by activating the AMPK/SIRT1 signaling pathway (Chen et al., 2024b). After intragastric administration of synthetic BBR lipid nanoparticles (Lip-BBR) (10 mg/kg b. wt per day for 14 weeks) activated LC3-II protein and AMPK/mTOR pathway and enhanced autophagy in hepatocytes of T2DM rats. It increase insulin synthesis, limit oxidative reactions to reduce endoplasmic reticulum stress, and have a certain protective effect on hepatocytes (Khater et al., 2023).

### 6.8 BBR ameliorates diabetic pulmonary injury

BBR, as the main active component of Coptis chinensis inflorescence extract, reduces the expression of inflammatory cytokines by activating the AMPK/NEU1 pathway and inhibiting the TGFβ1/Smad pathway, reverses EMT and reduces pathological damage in lung tissue (Wang et al., 2023).

### 6.9 BBR ameliorates diabetic reproductive dysfunction

**FIGURE 6:**
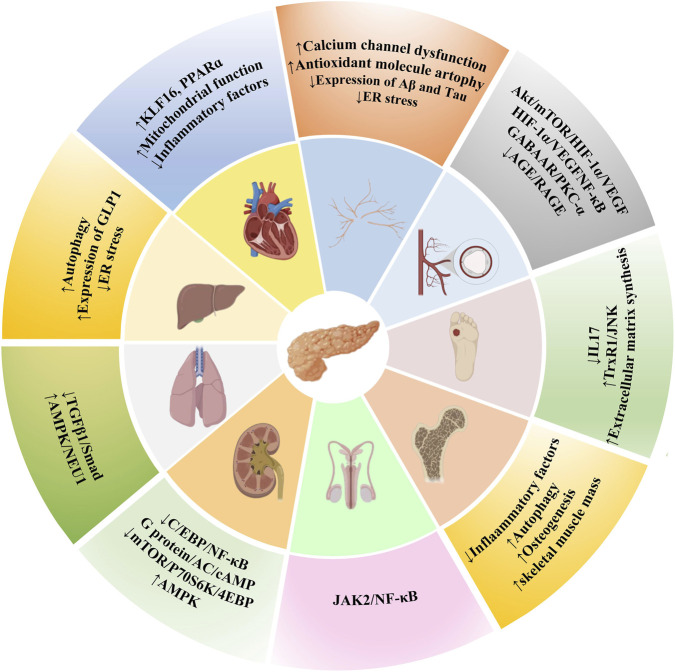
Protective effect of BBR on diabetic complications.

Diabetic rats decrease testicular weight and sperm viability, accompanied by impaired reproductive function. BBR have a protective effect on reproductive function in DM rats by reducing ROS production as well as testicular apoptosis through the JAK2/NFκB pathway (Song et al., 2020). It is also able to suppress apoptosis and improve erectile dysfunction in DM rats by suppressing the sphingosine kinase 1/sphingosine-1-phosphate/S1PR2 and MAPK pathways (Liu et al., 2022). Figure 6 and Table 3 summarize potential pathways which BBR exerted protective effects in the treatment of diabetic complications.

**TABLE 3:** BBR in the treatment of diabetes.

Component	Experimental model	Routes of administration	Pathologic changes	Critical proteins	References
BBR	db/db mice	Oral gavage	↓iron levels ↓lipid peroxidation accumulation ↑cell viability	NRF2	Zhang et al. (2025)
BBR	GK rats	Oral administration	↓FBG ↓HOMA-IR ↑GLP-1	Gut farnesoid X receptor	Zhao et al. (2021a)
BBR	HFD and STZ injection induced T2D model	—	↓IR ↓liver lipid accumulation ↓Injury to liver and pancreas	PPARγ-FGF21-GLUT2	Chen et al. (2023a)
BBR and HF	T2DM modle	Oral administration and low-dose intraperitoneal injection	↓MiR-204 levels ↑Insulin synthesis ↑SIRT1 expression	miR-204/SIRT1	Hou et al. (2020)
BBR	GDM	Oral administration	↓Body weight and fat mass ↓ mitochondrial respiratory impairment ↑β-cell function	—	Dang et al. (2023)
BBR nanoparticles	NMRI male	Intraperitoneal injection	↓Oxidative stress ↑Insulin secretion	Oxidative stress pathway	Lee and Lee (2022)
BBR	Feeding a high-sugar high-fat diet (HSHFD)	Oral gavage	↓IR ↓metabolic disorders	PI3K/AKT/TXNIP	Demir et al. (2021)
BBR	palmitate (PA)-induced hepatocyte IR model	Oral administration	↑Hepatic IR	SIRT1/Opa1	Shakeri et al. (2024)
BBR and probiotics	T2D patients	Oral administration	↓DCA transformation ↓Blood glucose ↑Microbial BA metabolism	DCA	Elkomy et al. (2022)
BBR	T2D patients	Oral administration	↓BCAAs, AAAs ↑Richness of intestinal microbiota ↑GLP-1	Multi-target compound action	Cheng et al. (2022)
BC	db/db mice	Oral gavage	↑Fecal DCA level ↑The colonic TGR5 ↑Serum GLP-1/-2 levels	TGR5-GLP	Wang et al. (2021a)
BBR	Feeding on GTGZ diet	Oral gavage	↓serum glycolipid level ↑intestinal barrier function ↑proliferation of beneficial microbiota	Gut microbiota-related tryptophan metabolites	Shu et al. (2021)
BBR	DN rat	Intraperitoneal injection	↓renal injury induced ↓ROS production	C/EBPb/Gas5/miR-18a-5p C/EBPb/PGC-1a	Bertoncello et al. (2024)
BBR	DM rat	Oral gavage	↑Glucose metabolism ↑Bone metabolism	ROS-mediated IRS-1	Londzin et al. (2022)
BBR	a rat model of T2DM	Oral gavage	↓Cell apoptosis ↑Autophagy activation	Autophagy of tendon fibroblasts	Liu et al. (2022)
BBR	STZ injection induced diabetic rats	Oral gavage	↓endothelial dysfunction ↓apoptosis	SPHK1/S1P/S1PR2 and MAPK	Ming et al. (2024)
BBR	Animal model of periodontitis	Oral gavage	↓M1polarization ↓Alveolar bone loss ↓proinflammatory cytokines ↑M2 polarization	NF-κB	Wang et al. (2021b)
BBR	STZ injection induced diabetic mice	Oral administration	↓Abnormal morphology of RPEs	CA1	Wang et al. (2021c)
BBR	Rat	Oral administration	↓AR activity ↓AOPP and AGEs levels ↑Soluble protein levels	AOPP and AGEs	Fang et al. (2022)
BBR	diabetic retinopathy rat model	Oral administration	↓RGCs apoptosis ↓GABA, PKC-α and Bcl-2 protein expression ↑The survival of RGCs	GABA-α receptor	Baum et al. (2021)
BBR	prediabetes rat model	Oral gavage	↓Oxidative stress ↓Apoptosis level of hippocampal tissue ↑Blood glucose ↑Lipid metabolism	—	Zhang et al. (2021a)
BBR	Alzheimer‘s diabetic rat	Intraperitoneal injection	↓TUNEL-positive cells ↓neuronal apoptosis	—	Zhang et al. (2021b)
BGB	HFD/STZ mice	Oral gavage	↓Tissue degeneration in myocardial infarction ↓Metabolic abnormalities	—	Xu et al. (2021b)
BBR	Male Dawn rat	Oral gavage	↓Fundic nerve dysfunction ↑The release of acetylcholine	Calcium channel	Zhang et al. (2022a)
BBR and Inulin	Patients with LADA	Oral administration	↑Metabolic	—	Ni et al. (2022)
BBR	Feeding a high-sugar high-fat diet (HSHFD) and STZ injection induced T2D model	Oral gavage	↓glucose uptake ↓abnormal renal function indicators	PI3K/Akt/AS160/GLUT1	Saxena et al. (2024)
BBR and HR	BBR and HR co-cultured with HK-2 cells	Oral administration	↓EMT ↓Renal interstitial fibrosis	NLRP3 inflammasome	Cai et al. (2024)
BBR	STZ injection induced diabetic mice	Oral gavage	↓ferroptosis ↓oxidative stress ↓methylation of KLF4 promoter methylation ↓expression of DNMT1 and DNMT2	KLF4	Zhang et al. (2021d)
BBR and Met	Db/db mice	Oral gavage	↓Inflammation ↑Lipolysis	Trib1	Wang et al. (2025)
BBR	BBR co-cultured with mice podocytes	*In vitro* co-culture	↓Podocyte apoptosis and injury ↑Podocyte viability ↑Autophagy	mTOR/P70S6K/4EBP1	Tannu et al. (2024)
BBR	HFD and STZ injection induced T2D model	Oral gavage	↓inflammasome activation ↑cardiac function	miR-18a-3p/Gsdmd	Cortez-Navarrete et al. (2023)
BBR	db/db mice	Oral gavage	↓inflammation and pyroptosis	mTOR/mtROS	Cole et al. (2021b)
BBR and HB	Wound healing in HFD and STZ injection induced diabetic rats	Local treatment	↓The IL-17 signaling pathway ↑Wound healing	IL-17 signaling pathway	Hu et al. (2023)
BBR	Wound healing in HFD and STZ injection induced diabetic rats	Local treatment	↓high-glucose-induced HaCaT cell injury ↑Wound healing ↑Extracellular matrix synthesis	TrxR1/JNK	Maity et al. (2022)
BBR and BSP	STZ injection induced diabetic mice	External use	↓oxidative stress ↓proinflammatory cytokines ↑antibacterial	—	Samadian et al. (2020)
ZnO-BBR/H	Wound healing in STZ injection induced diabetic rats	Local treatment	↑Wound healing rate ↑Anti-inflammatory and anti-oxidative stress abilities	Inflammatory pathways and antioxidant emergency pathways	Yang et al. (2024b)
BBR	HFD-fed mice	Oral gavage	↓IR ↓TG levels ↓hepatic steatosis	AKR1B10/PPAR	Wang et al. (2023)
BBR	db/db mice	—	↓liver injury ↓hepatic steatosis ↓oxidative stress ↑lipid metabolism	AMPK/SIRT1	Song et al. (2020)
Liposome-Encapsulated BBR	HFD and STZ injection induced diabetic rats	Oral administration	↓Insulin biosynthesis ↓Steatosis ↓Insulin biosynthesis ↑Lipid metabolism	AMPK/mTOR-Mediated Autophagy	Kodi et al. (2024)
BBR and LIN	HFD and STZ injection induced diabetic rats	Oral gavage	↓HDL-C ↑Lipid metabolism disorders ↑Serum TG, TC, and LDL-C levels	AMPK/NEU1	Babaei Khorzoughi et al. (2019)
BBR	STZ T2DM rats	Oral gavage	↑Reproductive function ↑Testis weight and ↑Sperm motility ↓Impairment of the seminiferous tubules	ROS/JAK2/NFκB	Akula et al. (2019)

## 7 BBR and metformin

Metformin is a foundational medication for type 2 diabetes, renowned for its significant blood glucose-lowering effects and ability to improve insulin resistance. Berberine (BBR) demonstrates similar efficacy; however, the mechanisms of action and primary focuses of BBR and metformin are not identical. When used in combination, they exhibit remarkable synergistic effects. Both can regulate glucose and lipid metabolism by activating the AMPK pathway. The combination of berberine and metformin not only produces superior hypoglycemic and insulin-sensitizing effects compared to monotherapy but also induces structural changes in the gut microbiota, such as significantly increasing the abundance of Proteobacteria and Verrucomicrobia (Lyu et al., 2022). In terms of lipid regulation, the combination therapy, at doses lower than the effective concentrations of either agent alone, synergistically downregulates the expression of key lipogenic transcription factor SREBP-1c and its downstream target gene FAS, thereby more potently inhibiting lipid synthesis (Kodi et al., 2024). Differences in their actions also exist. In a neuroinflammation model, metformin was able to ameliorate LPS-induced sickness-like behaviour and reduce oxidative stress, with effects superior to those of berberine, suggesting metformin’s potential advantage in anti-inflammation and neuroprotection (Babaei Khorzoughi et al., 2019). Both metformin and berberine can inhibit the proliferation of pancreatic cancer cells. Chemically modified berberine (NAX compounds) exhibit stronger activity, while metformin can directly suppress pancreatic cancer cell proliferation (Akula et al., 2019). The comparison of the effects of berberine (BBR) and metformin (MET), as well as the results of their combination therapy, will be presented in Table 4.

**TABLE 4:** Comparing the effects of berberine (BBR) and metformin (MET).

Comparison dimension	BBR	MET	BBR + MET
mechanism of action	Activating AMPK, regulating the intestinal flora, directly interacting with DNA/RNA, and influencing miRNA expression	Activate AMPK, inhibit hepatic gluconeogenesis, and regulate the intestinal flora	Enhance AMPK activation, uniquely regulate the microbiota, and alter drug pharmacokinetics
Blood sugar reduction and insulin sensitization	Effectively lowers blood sugar levels and improves insulin resistance, with an effect comparable to that of MET.	A first-line hypoglycemic drug that effectively improves insulin sensitivity	It can lower blood sugar levels more effectively than single drugs, and further improve insulin sensitivity (HOMA-IR)
Lipid regulation and anti-fatty liver disease	Inhibit the synthesis of lipids in liver cells	Inhibit the synthesis of lipids in liver cells	At extremely low concentrations, it can synergistically downregulate key lipid synthesis genes (SREBP-1c, FAS), thereby reducing the levels of cellular triglycerides and total lipids
Gut microbiota regulation	Regulate the bacterial community structure (such as reducing the F/B ratio)	Regulate the bacterial community structure (such as increasing the number of lactic acid bacteria)	This led to different changes in the bacterial community compared to the single drug treatment
antitumor activity	It inhibits the proliferation of pancreatic cancer cells, and its chemical modification products (NAX type) have stronger activity	Inhibit the proliferation of pancreatic cancer cells	Synergistic/Enhanced Inhibition: Certain BBR derivatives (such as NAX035, NAX060) when combined with MET can significantly enhance the inhibition of cancer cell proliferation, and the effect is influenced by the TP53 genotype

## 8 Novel antidiabetic strategy of BBR and its derivatives

BBR has a strong hypoglycemic effect, however its poor oral bioavailability hinders its further clinical application. Researchers have tried different strategies to address the limitations of BBR low bioavailability (Sharifi-Rad et al., 2021; Petrangolini et al., 2021; Zu et al., 2021). High-dose BBR treat diabetic symptoms, but it’s associated with significant gastrointestinal side effects (Kumaş et al., 2019) and cardiovascular system side effects (Martini et al., 2020).

Gastrointestinal side effects: BBR treatment induced gastrointestinal side effects. The study aims to control type 2 diabetes by oral probiotics or BBR to change the intestinal microbiota. More cases of gastrointestinal adverse effect (AE) cases occurred in both BBR arms and glycaemic control (Zhang et al., 2020).

Cardiovascular system side effects: Berberine induces developmental toxicity and pericardial edema in a time- and concentration dependent manner. Berberine can cause abnormalities in heart shape, manifested as stretching of the shape and separation of the endocardium/myocardium of the atrium; it can trigger abnormalities in heart function, leading to bradycardia, a reduction in cardiac output, the percentage of atrial shortening fraction, and atrial stroke volume; and it interferes with the angiogenic process (Martini et al., 2020).

Therefore, it is crucial to increase the bioavailability of BBR by different means.

### 8.1 Changing the formula to improve the antidiabetic effect of BBR

To enhance the effectiveness of BBR, an effective approach is to increase its bioavailability. Mixed BBR and sodium decanoate, an absorption enhancer, to prepare Huang-Gui solid dispersion (HGSD). HGSD shows a 3-fold increase in membrane permeability and a 5-fold increase in bioavailability. Oral administration of HGSD (100 mg/kg) significantly improved glucose and lipid metabolism in diabetic rats compared with pure BBR (100 mg/kg), BBR tablets (100 mg/kg), or metformin (300 mg/kg),this related to the inhibition of iPLA2/p38 MAPK pathway to decrease β-cell apoptosis (Bi et al., 2022; Zhaojie et al., 2014). The poor oral bioavailability of BBR is compromised by P-glycoprotein (P-gp), an active efflux protein that depletes adenosine triphosphate and extrudes BBR into the intestinal lumen, thereby limiting its absorption. Silymarin as a known P-gp antagonist, when used in combination with BBR effectively reduce glycated hemoglobin (Kwon et al., 2020; Di Pierro et al., 2013). Wang et al. prepare hexagonal plate-like granules containing BBR (BBR/MLDH), which also significantly improve solubility and oral availability and greatly enhance the hypoglycemic effect of BBR (Guo et al., 2020). Encapsulating BBR in liquid crystal nanoparticles (LCNs) of phytotriol showed more robust anti-inflammatory and antioxidant activity in lipopolysaccharide-induced RAW246.7 macrophages *in vitro* (Alnuqaydan et al., 2022). In addition, the use of hydrogen bonds or other non-covalent bonds to form drug co-crystals to improve the bioavailability, solubility, and stability of chemicals and reduce the administered dose or adverse effects has received much attention. Synthesis of RB from rosiglitazone with BBR at a ratio of 1:1 M markedly improved glucose and lipid metabolism and insulin resistance in diabetic mice (Shakeri et al., 2024). BBR-loaded bilosomes (BER-BLS) have also been prepared using a thin-film hydration strategy, which has higher stability and sustained release ability than BBR solution alone. Moreover, bioavailability increased 6.4-fold in diabetic rats (Elkomy et al., 2022).

### 8.2 Antidiabetic effect of BBR derivatives

A more promising strategy is to use BBR as a lead compound to obtain new antidiabetic agents by altering its structure. Molecular docking experiments had found that pseudo-Berberine (IMBY 53) had a lower affinity for P-gp, enhancing its blood sugar-lowering effect by prolonging its retention time in hepatocytes and muscle cells (Shan et al., 2013). Wang et al. designed and synthesized a series of derivatives of BBR, most of which had strong hypoglycemic activity. Among them, compound 20b, which has the strongest hypoglycemic effect, was 3.23-fold stronger than BBR and 1.39-fold stronger than metformin (Wang et al., 2019b). In derivatives synthesized by Nam et al. [WJCPR11-14 (Nam et al., 2021) and 1a-c, 2a-e, 3a-b (Nam et al., 2023)], WJCPR11 and 3b upregulated the expression of adipogenic genes and the levels of adiponectin (a unique marker of insulin sensitivity). More importantly, it did not cause cytotoxicity. Mechanistically, compound 3b attenuated phosphorylation of three MAPKs, and silicon molecular docking experiments also suggested that compound 3b may bind PPARγ. Bian et al. synthesized 16 BBR derivatives and assessed their structure-activity relationships through a mouse model of diabetes. The results indicate that substituents present on the A-ring appeared to be important for binding activity, and when the dimethoxy groups at C-2 and C-3 of BBR were replaced by other substituents, the binding affinity disappears. In addition, binding interactions were also influenced by N-7 potential and environment (Bian et al., 2006).

**FIGURE 7:**
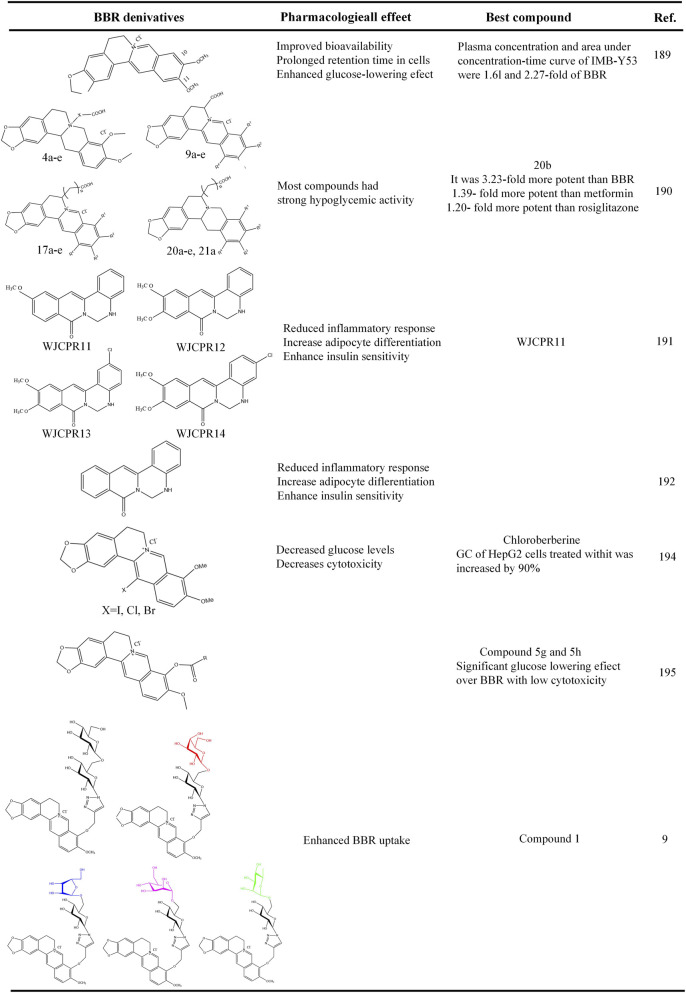
Protective effect of BBR derivatives in diabetes.

Ding et al. designed and synthesized halogenated BBR derivatives with bromine, iodine, and chlorine. Compared to the parent BBR, chloro and bromo BBR reduced glucose levels and cytotoxicity in HepG2 cells, with chloroberberine showing better efficacy (Ding et al., 2014). Introducing lipophilic moieties by modifying the lipophilic moiety structure of BBR was an effective way to enhance the antidiabetic activity. Zhang et al. synthesized 11 derivatives containing a 9-OH group. The results show that compounds 5a, 5g, and 5h had stronger hypoglycemic effects than BBR, while 5b, 5c, 5h, and 5i had relatively low cytotoxicity, and 5g and 5j had similar biocompatibility to BBR (Zhang et al., 2016). In addition to lipophilic groups, the carbohydrate part improved the bioavailability of the drug. Based on this, Wang et al. used classic “click” chemistry to modify the structure of BBR with disaccharides and tested five compounds in a zebrafish model. The results indicated that the modified BBR derivatives exhibited the highest anti-diabetic activity, greatly promoting the uptake of BBR by zebrafish larvae (Wang et al., 2020). The development of these lead compounds provided new ideas for the development of novel antidiabetic drugs. Figure 7 illustrates the protective effect of BBR derivatives in diabetes.

### 8.3 BBR self-assembly

BBR is an active component of traditional Chinese medicine with strong self-assembly ability and excellent pharmacological effects, and its research and development of carrier-free self-assembled nanomedicines has attracted much attention. BBR is self-assembled into nanoparticles (100–300 nm) with polysaccharides, flavonoids or proteins in traditional Chinese medicine compounds by hydrogen bonds and hydrophobic effects during decoction, which can significantly improve their water solubility (2.35-fold) and intestinal absorption rate (2.64-fold), while reducing the side effects of single components (such as gastrointestinal irritation) (Li et al., 2022c). This provides a brand-new perspective for BBR self-assembled nanoparticles in the study of diabetes treatment, and also closely combines the theory of traditional Chinese medicine with modern science and technology, so that traditional Chinese medicine is presented in the public eye with a brand-new face, opening an important way for new drug research and development.

## 9 Toxicity and safety

Although BBR is natural and widely used, its potential toxicity cannot be ignored. Despite the significant therapeutic potential of berberine in neurodegenerative diseases, its potential neurotoxicity cannot be overlooked. Specifically, in animal models, berberine at doses of 5–15 mg/kg has been shown to reduce the number of dopaminergic neurons in the substantia nigra and striatum, and inhibit dopamine synthesis. At the cellular level, micromolar concentrations (10–30 μM) of berberine not only enhance the toxicity of the neurotoxin 6-hydroxydopamine but also induce primary neuronal toxicity through a mitochondrial-dependent pathway and sensitize neurons to glutamate excitotoxicity. This toxicity can be alleviated by NMDA receptor antagonists (Ahmed et al., 2015). Some studies have shown that berberine has an inhibitory effect on immune response. Balb/c mice were intraperitoneally injected with berberine (5 and 10 mg/kg/day) for 14 consecutive days. The results showed that in the 10 mg/kg group, the numbers of white blood cells, neutrophils, and lymphocytes in the blood of mice significantly decreased, as did the numbers of CD19^+^ B cells, CD4^+^, and CD8^+^ T cells in the spleen (Mahmoudi et al., 2016).

As a safe medicinal plant component, berberine can significantly affect blood sugar levels, insulin resistance, blood lipids, inflammatory markers, colorectal adenomas and *Helicobacter pylori* infection. Berberine can improve various clinical outcomes (Li et al., 2023b). Panigrahi et al. conducted a clinical trial on patients with prediabetes. The treatment group was orally administered 500 mg of HIMABERB® three times a day, while the control group received a placebo. The results showed that at the midpoint and the end of the study, all blood sugar control indicators in the treatment group significantly decreased. Moreover, no serious adverse reactions, renal or liver toxicity were observed (Panigrahi and Mohanty, 2023). The main safety issue with berberine lies in the possibility of causing drug interactions.

## 10 Conclusion and prospects

In summary, BBR, as an important protoberberine alkaloid, was widely used in the treatment of diabetes. However, different separation and purification methods of BBR often result in low yields. Therefore, enhancing the research on the extraction and purification methods, as well as clarifying its structure-activity relationship at the primary and secondary structural levels, helped to understand its mechanism of action and develop more effective derivatives. To promote the basic research and clinical application, this article comprehensively summarized the pharmacological effects and clinical progress of BBR in treatment of diabetes and its complications. The interdisciplinary application had also sparked interest in the combined use of BBR or development of new derivatives for the treatment of diabetes. Although some clinical studies have been conducted, they are generally small in scale and rigorously designed. Therefore, it is urgent to initiate well-designed clinical trials to clarify the exact efficacy, optimal dose, and long-term safety of berberine in specific populations. In addition, standardized quality control methods and clear blood concentration-effect relationship were established to ensure the consistency of the efficacy of different batches of drugs and to achieve individualized medication. The intestinal absorption of berberine is weak and its bioavailability is low. Berberine is widely distributed in organs, but its concentration in blood is low. Future research should therefore focus on the development of novel delivery systems, such as formulations based on nanotechnology (e.g., liposomes, polymeric nanoparticles), self-microemulsion delivery systems, or prodrug strategies to improve their intestinal permeability and stability. BBR derivatives, with their excellent water solubility, lipophilicity, and higher oral bioavailability, significantly improved the pathological microenvironment of inflammation areas and reduced blood glucose levels. This provided a promising candidate drug for the treatment of metabolic diseases. Therefore, large-scale, long-term, multi-center clinical trials and the establishing of a standardized quality control method were still needed to evaluate the safety and efficacy of BBR and BBR derivatives in treatment.
